# Quality midwifery care during childbirth at a midwife obstetric unit: A qualitative study

**DOI:** 10.4102/curationis.v48i1.2773

**Published:** 2025-10-22

**Authors:** Sedeeka Martin, Gérard C. Filies, Anneline E. Robertson

**Affiliations:** 1 School of Nursing, Faculty of Community and Health Sciences, University of the Western Cape, Cape Town, South Africa; 2 Interprofessional Education Unit, Faculty of Community and Health Sciences, University of the Western Cape, Cape Town, South Africa

**Keywords:** quality, midwifery care, childbirth, midwife obstetric unit, women, midwives

## Abstract

**Background:**

Globally, there is an increasing focus on enhancing the quality of midwifery care. In public institutions, primary care midwifery is provided at a midwife obstetric unit (MOU). Midwives at MOUs are the first contact that pregnant women have when accessing midwifery care. There exists a notable disparity in the perception of quality midwifery care between midwives and women. In South Africa, there has been a scarcity of research examining the perspectives of midwives and women, regarding the quality of midwifery care provided during childbirth.

**Objectives:**

The objective of this study was to explore and describe women’s and midwives’ perceptions of the quality of midwifery care during childbirth, at a MOU in Cape Town in the Western Cape, South Africa.

**Method:**

A qualitative exploratory descriptive design was utilised. Four women and five midwives were selected through purposive sampling. Semi-structured interviews were undertaken, transcribed and analysed utilising Tesch’s eight-step approach to qualitative data analysis.

**Results:**

Three themes emerged: (1) understanding of quality midwifery care, (2) experiences of women during childbirth and (3) support received by women and midwives.

**Conclusion:**

The quality of midwifery care remains compromised within the MOU setting due to various challenges encompassing limitations of both human and physical resources. It is imperative to establish a shared understanding of what constitutes quality midwifery care, as women and midwives often perceive it differently.

**Contribution:**

Midwifery care during childbirth requires the engagement of all stakeholders, including women, to enhance the quality of midwifery care provided.

## Introduction

Contemporary maternity services prioritise delivering high-quality maternal care that ensures safety for all women. Additionally, midwives are expected to exhibit respect and genuine interest in women, approaching them with a non-judgemental attitude (Martin & Filies 2018; Republic of South Africa [RSA], Department of Health [DoH] 2016). Globally, many women experience disrespectful, abusive or neglectful care during childbirth in facilities (World Health Organization [WHO], 2014). The WHO recommendations on intrapartum care for a positive childbirth experience highlights respectful care as a key recommendation, and respectful maternity care is emerging as an essential concept for ensuring the rights and safety of women during labour (WHO 2018:3).

Quality maternity care includes several key components: competent and well-trained midwives who receive continuous education, women-centred care that promotes clear communication and respects women’s backgrounds while supporting their autonomy in decision making. It also requires a work environment with adequate equipment, resources and psychological support for women, and staff who comply with set guidelines and quality assurance measures (Afulani et al. 2017; Bohren et al. 2015; WHO 2016).

The consequences of poor-quality care directly impact maternal and neonatal mortality and morbidity as it increases the risk of maternal and neonatal complications. The delay in management of complications can lead to significant morbidity or death, psychological trauma and a loss of confidence in healthcare provision by the public (Afulani et al. 2017; Bohren et al. 2015).

The South African Nursing Council (SANC) Scope of Practice, also known as Regulation 2598, provides guidelines that direct midwives in delivering quality care to pregnant and birthing women in South Africa (Martin & Filies 2018; SANC R2598 of 1984). In 2014, the Western Cape Department of Health (WCDoH) published the Patient Centred Maternity Care (PCMC) code, which complements the SANC regulations in ensuring the delivery of quality maternity care to women during childbirth; this PCMC code represents a fundamental principle within the strategic plan of the WCDoH’s strategy towards Healthcare 2030. It also underscores the Western Cape’s dedication to patient-centred maternity care (WCDoH 2014).

O’Donnell et al. (2014) discovered that understanding the essence of quality midwifery care is pivotal for its provision. Their research study revealed significant disparities in perceptions between midwives and women receiving maternity care. The women regarded having a good relationship with the midwife as important, while midwives considered having the necessary resources available to deliver quality care as important.

During childbirth, women rely heavily on midwives for assistance, rendering them especially vulnerable during the intrapartum period (Sacks & Kinney 2015). Bhattacharyya et al. (2018) emphasise that childbirth is a profoundly emotional event for women, underscoring the explicit aim of midwives to ensure a positive childbirth experience for each woman (Martin & Filies 2018). As women hold distinct expectations regarding the quality of midwifery care at childbirth facilities, aligning these expectations with midwives’ perspectives is imperative for enhancing the quality of childbirth care. The utilisation of services and maternal health outcomes is influenced not only by the quality of care provided but also by women’s experiences of that care (Martin & Filies 2018; Mgawadere et al. 2019; Tiruneh et al. 2021). Consequently, while midwives may consider the care provided during childbirth to adhere to established standards, it may be perceived as unsatisfactory by women, their families and the community (Norhayati et al. 2017; Lohmann, Mattern & Ayerle 2018).

In South Africa, many birthing women face a lack of empathy and endure disrespectful and abusive care during the intrapartum period (Oosthuizen et al. 2019). In response, a mixed-methods intervention study was undertaken in 2019 in Tshwane, South Africa, involving 10 midwife-led obstetric units (MOUs). The aim of the intervention was to promote sustainable improvement in obstetric care practices and foster respectful, competent midwife-led quality care during labour and delivery in MOUs. The objective of this context-specific intervention package was to address the intricate relationship between preventable maternal and perinatal mortality and morbidity and issues related to poor clinical governance and supervision in midwife-led labour units. Hence, the focus of the results was to determine whether there was a reduction in mortality and morbidity. The authors argued that if respectful care is provided, it will result in better perinatal outcomes and subsequently quality midwifery care (Oosthuizen et al. 2019):

In the Western Cape, there has been a scarcity of research investigating the perceptions of midwives and women regarding quality midwifery care. In light of this gap, this study endeavours to delve into and elucidate the perceptions of women and midwives concerning quality midwifery care during childbirth at one of the MOUs in the Western Cape (Martin & Filies 2018). The findings of this study have the potential to offer valuable insights to policymakers and maternity health providers, particularly midwives, aiding them in comprehending whether the care they deliver, particularly within MOUs, aligns with the expectations of birthing women (Martin & Filies 2018).

### Problem statement

In 2014, the WCDoH’s strategy for the PCMC code was to deliver high-quality maternity care to birthing women. In 2018, the WHO published the recommendations for intrapartum care for a positive childbirth experience, which highlight respectful care as a key recommendation for ensuring the rights and safety of women during labour (WHO 2018). Although the WCDoH’s strategy and the publication of WHO’s recommendations were designed to strengthen the quality of care women receive during childbirth, frequent public reports of dissatisfaction suggest ongoing challenges. During this period, women are most vulnerable and public complaints are associated with poor communication, neglect, insufficient emotional support and in some cases reports of disrespectful and abusive treatment by midwives (Fraser, Cooper & Nolte 2010). A study by Malatji and Madiba (2020) revealed that disrespect and abuse of women during childbirth are common in South African MOUs. The midwives frequently provide disrespectful care throughout the childbirth process. Implementation of a policy framework is insufficient to ensure quality care during childbirth. Instead, these issues identify institutional shortcomings in areas of staff training, on-site quality assurance and institutional accountability (Afulani et al. 2017).

This study aimed to investigate and outline the perceptions of women and midwives regarding the quality of midwifery care offered during childbirth at a specific MOU in Cape Town, Western Cape. The objectives included examining and delineating women’s perceptions of the care received during childbirth, as well as exploring and detailing midwives’ perspectives on the care provided during childbirth (Martin & Filies 2018).

## Research methods and design

### Design

A qualitative, descriptive, and explorative research design was used for this research study. The design facilitated women and midwives in expressing their views on the quality of midwifery care provided during childbirth at MOUs in the Western Cape. Individual semi-structured interviews were conducted to collect data (Martin & Filies 2018).

### Setting

The study was conducted at an MOU situated in Cape Town, Western Cape. This facility is part of the Metropole District Health Services (MDHS), which oversees health facilities spanning from Cape Point to Atlantis across Cape Town. The MDHS is organised into eight sub-structures, each comprising two sub-districts, with the grouping based on population size, geographical area and drainage areas (MDHS 2004; Martin & Filies 2018). The MOU falls under the Klipfontein-Mitchells Plain sub-structure. The MOU serves the largest population and has the highest childbirth rate. As per the City of Cape Town Census in 2011, the population served by the MOU is 507 237, with the subsequent census scheduled for 2022 (Martin & Filies 2018; Statistics and Population Census 2013). The Census 2022 information was released on 10 October 2023 and only covers high-level reports and selected indicators at the national, provincial, district and local municipality levels. The census datasets, including sub-metro data and other standard indicators, will only be available in later phases.

### Study population and sampling strategy

The MOU has eight midwives allocated to the day and night shifts in the labour ward. Participants for the study were chosen using a purposive sampling approach. Four women who had experienced uncomplicated births and were within seven days post-delivery were selected. The parity of the women ranged from primipara to parity four. Parity refers to the number of viable births (Fraser et al. 2010). Additionally, five midwives who were permanent employees at the MOU and had worked in the labour ward for a minimum of 12 months were chosen (Martin & Filies 2018).

### Data collection

A semi-structured interview guide was developed in both English and Afrikaans, although English was the preferred language for both women and midwives. The guide was created by the first author in collaboration with the second author, aligning with the research objectives. Data saturation was reached after the sixth interview, but the first author conducted additional interviews until the ninth participant to ensure comprehensive data collection. The first author, serving as a clinical supervisor at a university, had no prior interaction with the participants.

After obtaining permission from the DoH, the first author initiated contact with the operational manager via email to provide study details and request access to the MOU. Subsequently, informational posters containing study details and the first author’s contact information were prominently displayed in waiting areas, complemented by the distribution of flyers to women. Group sessions were conducted by the first author in waiting areas to inform postnatal women about the study. Utilising the MOU birth register, contact information of women who had uncomplicated deliveries within the past seven days was obtained. Midwives were approached during both day and night shifts to inform them about the research study. All potential participants were contacted by the researcher to schedule interviews at a suitable time and location (Martin & Filies 2018). A pilot interview was conducted with a midwife, adhering to the inclusion criteria, to refine the interview guide and enhance the first author’s interviewing skills. The insights gained from the pilot interview were valuable and were integrated into the data analysis. Interviews took place in a private room within the MOU, with a notice outside the door indicating ‘Do not disturb, Interview in Progress’ to prevent disruptions (Martin & Filies 2018).

Before commencing the interviews, the first author informed the participants about the study and confirmed their willingness to participate, including consenting to have the interview audio recorded. Participants were informed that their involvement was voluntary and that they could withdraw at any point without consequences. Additionally, participants were assured that their identities would remain anonymous during data analysis. The interviews lasted between 45 min and 60 min. Some women were interviewed while holding their babies, which influenced the length and flow of the interviews. This occasionally led to interruptions as their infant’s needs were prioritised.

### Data analysis

The interviews underwent transcription and analysis following Tesch’s eight-step model (Creswell 2009). Both the first and second authors transcribed the audio recordings. The first author listened to the interviews, thoroughly read the transcripts, and documented emerging ideas directly onto them. Each transcript was numbered, and pairs of transcripts were reviewed, with relevant data noted down. Positive and negative perceptions were distinguished using different colour pens. This process continued until all transcripts were reviewed. Similar topics were identified, listed and clustered together. Major, unique and residual topics were grouped into columns. These topics were then condensed into codes by referring to the data. This process facilitated the identification of new categories and codes. Related topics were grouped together, and descriptive terms were assigned to form themes and subthemes (Martin & Filies 2018).

### Trustworthiness

Trustworthiness in this study was maintained through adherence to the principles outlined by Lincoln and Guba (1985), as cited by Cypress (2017), including credibility, transferability, dependability, and confirmability. Purposive sampling ensured that participants could offer detailed insights, allowing their own words to be directly quoted. Participants were strictly selected based on predefined inclusion criteria. Consistency was ensured by employing the same semi-structured interview guide for all participants. Probes were used during interviews to gather detailed data, while reflective questioning enabled the researcher to delve deeper into participants’ responses. Peer review was conducted by the second author throughout the study to enhance rigour and validity.

### Ethical considerations

Ethical permission was obtained to conduct the study from the University of the Western Cape Research Ethics Committee (reference number: 15/7/265), and permission was obtained from the WCDoH. Finally, institutional permission was granted by the Primary Health Manager of the Mitchells Plain Community Health Centre (Martin & Filies 2018). Informed written consent was obtained from the participants after they read the participant information sheet, which clearly explained the purpose of the research, selection method, data collection procedure and benefits. The participants were assured that participation was voluntary and that they could withdraw from the study at any time (Martin & Filies 2018). Acquiring informed written consent helped ensure that participants willingly participated in the research without coercion. The researcher acknowledged that discussing past experiences may have been distressing to some participants. Should a woman become distressed, she would be referred to the mental health services at the facility. No woman required a referral. Confidentiality and anonymity were guaranteed by ensuring that data obtained would not be shared with anyone other than the authors.

## Results

Three themes and nine subthemes emerged from the data and are depicted in Table 1.

**TABLE 1:** Themes and subthemes.

Theme	Subthemes
1 Understanding of quality midwifery care	1.1 Women being informed. 1.2 Women receiving pain relief. 1.3 Midwives providing a safe delivery.
2 Experiences of women during childbirth	2.1 Women reporting being verbally abused. 2.2 Women describing uncaring behaviour. 2.3 Women reporting no care provided.
3 Support received by women and midwives	3.1 Women receiving support from midwives. 3.2 Women receiving support from a companion. 3.3 Midwives receiving support from management.

*Source:* Martin, S. & Filies, G.C., 2018, ‘Quality care during childbirth at a midwife obstetric unit in Cape Town, Western Cape: Women and midwives’ perceptions’, Magister Curationis thesis, University of the Western Cape, viewed 31 March 2024, from https://uwcscholar.uwc.ac.za/items/a9bb8cf6-a4b9-4bca-855d-64c80f93ec04/full

### Demographic characteristics of participants

Nine participants were interviewed in total: four women and five midwives. The ages of the women ranged between 17 and 32 years. The five midwives’ ages ranged between 25 and 58 years. Two of the midwives had an advanced midwifery qualification with 10 years and 30 years’ experience respectively. The other three midwives had a basic midwifery qualification and had less than 5 years’ experience.

### Findings

Each theme is presented with the perceptions of both midwives and women, allowing for a discussion of their experiences.

#### Theme 1: Understanding of quality midwifery care

Three subthemes emerged: women being informed, women receiving pain relief and midwives providing a safe delivery.

**Subtheme 1.1:** Women being informed: There was a discrepancy in perceptions regarding the adequacy of information provided to women for childbirth preparation. Some women expressed dissatisfaction, feeling that the information they received was inadequate and did not adequately prepare them for the challenges they encountered during labour. Conversely, midwives reported that they provided sufficient information to women during labour to assist them through the birthing process (Martin & Filies 2018):

‘I was here for the whole weekend basically. I went back and forth, went home, came back, went home, came back and I gave birth this morning at 4 o’clock … it was my first time; I didn’t know what to expect or you know if they are doing their job properly.’ (Woman four, 17 years old, primipara)

Midwives disagreed with the notion, asserting that women are indeed provided with adequate information during childbirth:

‘Every time you examine her, me personally as a midwife, I would tell the patient you are so far dilated, and this is what you can expect.’ (Midwife one, 25 years old, two years’ experience)

Another midwife agreed:

‘The thing that we do normally is to explain to the patient what to expect.’ (Midwife three, 27 years old, four years’ experience)

**Subtheme 1.2:** Women receiving pain relief: Among the women interviewed, only one considered pain relief to be a crucial aspect of quality care during childbirth (Martin & Filies 2018):

‘What I didn’t like is when you have pain here, they say you have to walk around I don’t think it is okay, you must walk around.’ (Woman three, 21 years old, parity two)

Midwives were inconsistent in administering pain relief to women, as most of the midwives opted for non-pharmaceutical methods instead (Martin & Filies 2018):

‘Pain relief is not practiced, and I mean the pain is so you can’t handle the pain and with the youngsters, you might want to consider at least them you might not give everybody, but that might open a can of worms: why is that mother getting and you can’t get pain relief … Because we don’t have the manpower to monitor the baby closely, and after that, the baby—because of the respiratory distress—you might have to spend more time post-delivery with the baby.’ (Midwife two, 37 years old, one year experience)

**Subtheme 1.3:** Midwives providing a safe delivery: All the midwives interviewed conveyed genuine concern for the well-being of women, ensuring safe delivery (Martin & Filies 2018). When the midwife was asked what she understood by quality midwifery care, the midwife replied:

‘… as the midwife to make sure that the baby is delivered safely and that the baby gets the care that the baby needs and the mother gets the post-delivery.’ (Midwife two, 37 years old, one year experience)

#### Theme 2: Experiences of women during childbirth

The women experienced positive and negative experiences, which contributed to the provision of quality midwifery care. The negative experiences included women reporting being verbally abused, women describing uncaring behaviour and women reporting a lack of care. The positive experiences included women receiving support from midwives and women receiving support from a companion.

**Subtheme 2.1:** Women reporting being verbally abused: One of the women reported feeling discriminated against by midwives because of her preference for the place of childbirth (Martin & Filies 2018):

‘… they were complaining that I am from Guguletu and enquired about why I am not using the clinic there and come here. So every time when I scream, you could hear them saying “you make us work, work hard because you leave your Gugulethu and come here”…’ (Woman two, 27 years old, parity two)

After thorough probing, the woman elaborated on the verbal abuse she experienced during childbirth:

‘The lady there was shouting at me … “Shut up! This is not Guguletu, you see … you don’t have to cry, you have to shut your mouth, open your legs, push! I’m not going to tell you anything now, I’m waiting for you!” … I don’t know— maybe she was helping me, maybe I wasn’t doing exactly what they expect of me.’ (Woman two, 27 years old, parity two)

**Subtheme 2.2:** Women describing uncaring behaviour: The women expressed that they were frustrated at being ignored and ‘not being believed’ regarding their complaints:

‘I was alone: no students, no sisters, nobody to help me. I was actually shouting for help, but nobody was there.’ (Woman one, 32 years old, parity four)

‘I was telling them that I am feeling faint; it was like I’m lying. They told me everything is fine, they told me my blood pressure and sugar is fine but for them it was like I’m not right in my head.’ (Woman three, 27 years old, parity two)

**Subtheme 2.3:** Women reporting no care provided: The women expressed that they were unheard or were abandoned, leading to feelings of fear or anxiety because of limited interaction with midwives:

‘So they told me they were busy, the sister them [*the midwives*].’ (Woman one, 32 years old, parity four)

‘There were other Sisters [*midwives*] that would just walk past and didn’t take note … or maybe they were busy with someone else or something.’ (Woman four, 17 years old, primipara)

#### Theme 3: Support received by women and midwives

The women received ineffective support from the midwives. The women relayed that they were not allowed to have a companion with them, and the midwives did not receive effective support from management.

**Subtheme 3.1:** Women receiving support from midwives: Women explained that there was a lack of support from:

‘I was alone: no students, no sisters, nobody to help me. I was actually shouting for help but nobody was there.’ (Woman one, 32 years old, parity four)

‘The one [*midwife*] came sometimes and left, and the other one was there to help me and she was good, she was great helping me get through it.’ (Woman four, 17 years old, Primipara)

**Subtheme 3.2:** Women receiving support from a companion: Women expressed the need to have a companion during childbirth, and midwives were inconsistent in allowing companions to support birthing women.

Midwife one explained that because of the facility’s infrastructure, birth companions are sometimes not allowed:

‘we allow patients to have companions, doulas, but because our facility is so small, and especially in the antenatal ward, that is like limited, so we don’t allow the doulas to be in with them from 1 cm to 3 cm; but as soon as they hit 4 cm and we put them in the labour ward and we make sure the doula is there to comfort them and to support them during childbirth.’ (Midwife one, 25 years old, two years’ experience)

A woman disputed this by saying:

‘I asked if my husband can come in; they said no, which is not a nice thing because you need someone with you at that time … actually the pain was so bad that I grabbed the sister’s hand, where my husband could have been there to support me.’ (Woman three, 21 years old, parity two)

**Subtheme 3.3:** Midwives receiving support from management: Midwives reported the clear impact of the lack of adequate, skilled staff on their performance and ability to meet professional standards and expectations. They described the circumstances that affected the care they could render:

‘… we don’t have a lot of sisters in the labour ward, so each and every patient can’t have a sister looking them.’ (Midwife three, 27 years old, four years’ experience)

Another midwife further explained:

‘Unfortunately, sometimes we are short-staffed; unfortunately, it’s full we can’t we try our best but we can’t always get to everything.’ (Midwife four, 37 years old, 10 years’ experience)

Midwives explained that they felt unappreciated by management, and it affected the level of care they rendered. Midwife one was very emotional during the interview and started crying when she spoke about the lack of support from management:

‘… you come to work; it’s like you feel incompetent because management doesn’t appreciate you; you just have to do what they tell you to do and if you raise concerns, it’s never met.’ (Midwife one, 25 years old, two years’ experience)

‘You as one person can’t manage all the patients, so there is a postnatal section that is being neglected and I think that the care of this facility is down because it is not properly staffed. You can’t say that one person because it is all, all of us are affected by this.’ (Midwife two, 37 years old, four years’ experience)

‘… at the end of the day, it is an administrative thing; with short staff, we can only say, we are struggling, we are tired, we are burnt out and from there it goes to management and management decides. I think they try to relieve it by getting agency people but the problem is the agency people can say they not coming tonight, then we are stuck. You as the permanent staff member, cannot say you not feeling well, because then there will be nobody on duty [*shrugging her shoulders*].’ (Midwife four, 37 years old, ten years’ experience)

## Discussion

The study findings unveiled disparities between women and midwives in their perceptions of quality midwifery care (Martin & Filies 2018).

Women emphasised the importance of receiving support during childbirth, being informed about their progress of labour and being treated with respect as quality midwifery care. This is reiterated in a study performed by Renfrew et al. (2014). The study findings revealed that women desire healthcare professionals who blend clinical expertise with interpersonal and cultural competence.

Women were able to articulate their perceptions of the meaning of quality midwifery care, as well as instances of inadequate or unsatisfactory care. However, they lacked awareness regarding the expected standards of care at the MOU and the responsibilities of midwives, which aligns with the findings of previous studies (Ahmed, Mahimbo & Dawson 2021; Martin & Filies 2018).

In a qualitative study by Larsson et al. (2019), women who harboured fears about childbirth received counselling from experienced midwives. These women reported that the counselling sessions, which provided information and knowledge, bolstered their confidence and imparted a sense of calm and readiness. Consequently, their childbirth experience was positively influenced, as they felt safer because of the professional support received during labour.

In the systematic review conducted by Bohren et al. (2017), it was affirmed that offering consistent information about the progression of labour empowered women, granting them a sense of personal control over the childbirth process, which is an essential predictor of childbirth satisfaction. This empowerment enabled women to emotionally prepare for the unpredictable journey of labour and delivery.

Midwives expressed genuine concern for women during childbirth, viewing ensuring a safe delivery as indicative of quality midwifery care. Despite their familiarity with caregiver duties and established protocols for safe care, midwives face daily challenges that they believe could impact the quality of care provided (Martin & Filies 2018). Health workers’ perceptions of insufficient staffing or time to carry out their responsibilities can significantly affect motivation and retention, leading to substandard care and negative attitudes towards women. This, in turn, may dissuade women from seeking maternity care (Bradley et al. 2015; Martin & Filies 2018).

Both the women and midwives had similar perceptions of what constitutes poor-quality care. This included inadequate staff to care for women in labour, verbal abuse and lack of communication between women and midwives, which were described as examples of poor-quality midwifery care.

This study also highlighted the urgent necessity to educate women and their family members about their entitlement to respectful care, empowering them to report and challenge any instances of disrespectful and abusive practices (Martin & Filies 2018). According to the Center for Reproductive Health (2018), respectful and safe maternity health care is a human right. Every women has a right to life, health, equality and non discrimination, this includes a safe pregnancy and childbirth.

The Center further asserts that governments have a responsibility to safeguard these rights by establishing conditions that promote the well-being of women, healthy pregnancies and safe births. Violations of fundamental human rights occur when pregnant and birthing women experience avoidable suffering, such as death, illness, injury, mistreatment, abuse, discrimination, and lack of information (Center for Reproductive Health 2018).

Another concerning issue which was highlighted by the findings of this study is the ever-present problem of staff shortages in South African labour wards. Midwives commented that staff shortages make it very difficult for them to render quality midwifery care. There are currently no existing staffing norms for the maternity wards in South Africa; however, in 2019, the International Federation of Gynaecology and Obstetrics released a position statement on Staffing Requirements for Delivery Care, With Special Reference To Low- and Middle-income Countries (Stones et al. 2019). The FIGO recommends that staffing should be based on the number of births the unit conducts a year. In addition, the FIGO recommends that the midwife-to-birthing-woman ratio should be based on the stage of labour women are in. This position statement recommends that during the active phase of labour, the ratio is one midwife for every two women. The recommended ratio for the second and third stages is two midwives for every birthing woman. This is to ensure quality care for both the mother and the neonate. Thereafter, in the postnatal ward, one midwife is recommended for every four women (Stones et al. 2019).

Furthermore, this study emphasised the significance of sensitising and training midwives in delivering respectful maternity care. Ending disrespect and abuse during childbirth requires the engagement of all stakeholders, including women, in endeavours to enhance the quality of midwifery care, as emphasised by the WHO (Martin & Filies 2018).

### Recommendations

#### Policy making

It is recommended that policymakers consider that the preparation for childbirth should commence during the antenatal period. This education should include information on non-pharmacological pain relief methods and childbirth preparation to ensure women are well prepared for labour (Martin & Filies 2018).

Each institution must ensure that midwives are well-informed about the formal measures in place to uphold professional accountability for patient safety and well-being. Continuous professional development on respectful midwifery care should be mandated for all midwives, aiming to eliminate unacceptable behaviour and negative attitudes. Additionally, a quality improvement programme should be developed and implemented to improve the nature, scope and quality of midwifery care provided in the unit (Martin & Filies 2018).

Staffing levels need to be adjusted based on the patient volume to ensure that the quality of care delivered is not impacted as a result of increased workload (Martin & Filies 2018).

Policymakers should acknowledge the importance of women having a companion during childbirth. This should be integrated into antenatal education, enabling women and their companions to make adequate preparations to enhance their birth experience (Martin & Filies 2018).

#### Midwifery education

Nursing programmes must incorporate antenatal and childbirth education, including respectful care, pain relief options and communication strategies.

When educating midwives, the focus must be on maintaining professional accountability, patient safety and practising within an ethical framework.

In-service training must be implemented to promote respectful maternal care and patient-centred practices.

#### Practice

Midwives must adhere to the ethical and respectful standards of care to avoid any negative attitudes or behaviours.

A formal quality improvement programme should be developed and implemented to assess the nature, scope and intensity of midwifery care provided in the unit.

#### Research

Further research should focus on evaluating the impact of implementing childbirth education and respectful care protocols on maternal outcomes and quality of care.

In addition, there is a need for intervention studies to investigate how ongoing professional development influences midwife performance, patient safety and labour outcomes.

### Limitations

This study is confined to a single MOU in the Western Cape, limiting its generalisability. The relatively small sample size of the study population also impacts the transferability of the findings. However, the integration of field notes and interview data offers a comprehensive understanding of women and midwives’ perceptions of quality care during childbirth (Martin & Filies 2018).

The study exclusively interviewed women who delivered their babies during the day and midwives on duty during the day shift. As a result, the findings cannot be generalised to all women and midwives at the MOU (Martin & Filies 2018).

## Conclusion

The aim of this study was to explore the perceptions of quality midwifery care during childbirth among women and midwives. The findings revealed significant challenges in the MOU setting, indicating a compromise in the provision of quality midwifery care. A notable discrepancy was observed between the perspectives of women and midwives regarding what constitutes quality care. Women emphasised the importance of receiving support, being informed about their progress in labour, and being treated with respect as essential aspects of quality midwifery care. Conversely, midwives primarily viewed ensuring a safe delivery as the epitome of quality care. It is imperative to foster a mutual understanding of quality midwifery care across all midwifery care settings to address these disparities and enhance the overall quality of midwifery care provided.
